# IQGAP1 Interaction with RHO Family Proteins Revisited: KINETIC AND EQUILIBRIUM EVIDENCE FOR MULTIPLE DISTINCT BINDING SITES*[Fn FN2]

**DOI:** 10.1074/jbc.M116.752121

**Published:** 2016-11-04

**Authors:** Kazem Nouri, Eyad K. Fansa, Ehsan Amin, Radovan Dvorsky, Lothar Gremer, Dieter Willbold, Lutz Schmitt, David J. Timson, Mohammad R. Ahmadian

**Affiliations:** From the ‡ Institute of Biochemistry and Molecular Biology II, Medical Faculty of the Heinrich-Heine University, 40225 Düsseldorf, Germany,; the § Institute of Physical Biology, Heinrich-Heine University, 40225 Düsseldorf, Germany,; ¶ Forschungszentrum Jülich, ICS-6, 52428 Jülich, Germany,; the ‖ Institute of Biochemistry, Heinrich-Heine University, 40225 Düsseldorf, Germany, and; the ** School of Pharmacy and Biomolecular Sciences, University of Brighton, Huxley Building, Lewes Road, Brighton BN2 4GJ, United Kingdom

**Keywords:** CDC42, fluorescence anisotropy, GTPase activating protein (GAP), protein-protein interaction, Ras homolog gene family, member A (RHOA), Ras-related C3 botulinum toxin substrate 1 (Rac1), Rho (Rho GTPase), signal transduction, IQGAP1, binding affinity

## Abstract

IQ motif-containing GTPase activating protein 1 (IQGAP1) plays a central role in the physical assembly of relevant signaling networks that are responsible for various cellular processes, including cell adhesion, polarity, and transmigration. The RHO family proteins CDC42 and RAC1 have been shown to mainly interact with the GAP-related domain (GRD) of IQGAP1. However, the role of its RASGAP C-terminal (RGCT) and C-terminal domains in the interactions with RHO proteins has remained obscure. Here, we demonstrate that IQGAP1 interactions with RHO proteins underlie a multiple-step binding mechanism: (i) a high affinity, GTP-dependent binding of RGCT to the switch regions of CDC42 or RAC1 and (ii) a very low affinity binding of GRD and a C terminus adjacent to the switch regions. These data were confirmed by phosphomimetic mutation of serine 1443 to glutamate within RGCT, which led to a significant reduction of IQGAP1 affinity for CDC42 and RAC1, clearly disclosing the critical role of RGCT for these interactions. Unlike CDC42, an extremely low affinity was determined for the RAC1-GRD interaction, suggesting that the molecular nature of IQGAP1 interaction with CDC42 partially differs from that of RAC1. Our study provides new insights into the interaction characteristics of IQGAP1 with RHO family proteins and highlights the complementary importance of kinetic and equilibrium analyses. We propose that the ability of IQGAP1 to interact with RHO proteins is based on a multiple-step binding process, which is a prerequisite for the dynamic functions of IQGAP1 as a scaffolding protein and a critical mechanism in temporal regulation and integration of IQGAP1-mediated cellular responses.

## Introduction

The RHO family proteins are critical regulators of many diverse cellular functions (1). They share two common functional characteristics, potential membrane anchorage, and an on/off switch cycle (2). RHO protein function is dependent on the guanine nucleotide binding (G) domain that contains the principal GDP/GTP binding pocket and presents, depending on its nucleotide-bound state, various contact sites for regulators and effectors (2). Membrane-associated RHO proteins act, with some exceptions (3), as molecular switches by cycling between an inactive GDP-bound state and an active GTP-bound state. This cycle underlies two critical intrinsic reactions, the GDP/GTP exchange and GTP hydrolysis (3), and is controlled by three main classes of regulatory proteins (2), guanine nucleotide dissociation inhibitors (GDIs)^3^ (4), guanine nucleotide exchange factors (GEFs) (5), and GTPase activating proteins (GAPs) (6). The formation of the active, GTP-bound state of RHO proteins is accompanied by a conformational change in two regions known as switch I and II (2). These regions provide a platform for the selective interaction with structurally and functionally diverse effectors (2, 7, 8), *e.g.* p21-activated kinase 1 (PAK1) (9), Wiskott-Aldrich syndrome proteins (WASP) (10), p67^phox^, a member of the NADPH oxidase family (11), and semaphorin receptor Plexin B1 (12, 13) as well as the IQ motif-containing GTPase activating proteins (IQGAPs) (14, 15).

**FIGURE 1.**
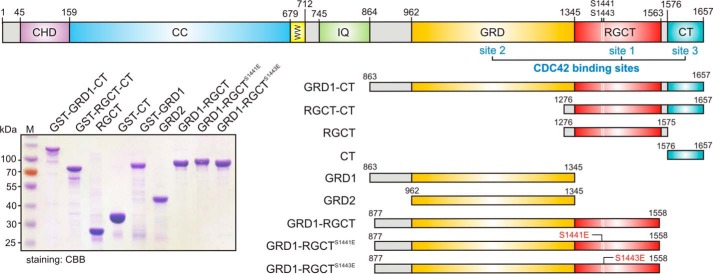
**Schematic representation of domain organization, various constructs, and proteins of IQGAP1.** Shown is IQGAP1 domain organization along with the PKCϵ phosphorylation sites Ser-1441 and Ser-1443, constructs, and proteins relevant to this study. Coomassie Brilliant Blue (*CBB*)-stained SDS-PAGE (12.5%) of purified IQGAP1 proteins used in this study is shown. *CHD*, calponin homology domain; *CC*, coiled-coil repeat region; *WW*, tryptophan-containing proline-rich motif-binding region; *IQ*, four isoleucine/glutamine-containing motifs.

IQGAP1 is a ubiquitously expressed scaffold protein involved in a variety of cellular processes, such as cell motility, cell-cell adhesion, protein trafficking, transcription, neoplasia, and microbial pathogenesis (14–17). A prerequisite to achieve these functions is association of a multitude of signaling molecules, *e.g.* calmodulin, kinases, and GTPases such as CDC42 and RAC1 (18–21). Distinct domains of IQGAP1, include an N-terminal calponin homology domain (CHD), a coiled-coil repeat region (CC), a tryptophan-containing proline-rich motif-binding region (WW), four isoleucine/glutamine-containing motifs (IQ), a RAS GAP-related domain (GRD), a RASGAP C-terminal domain (RGCT), and an extreme C-terminal domain (CT) (Fig. 1).

Work from several laboratories has shown that the C-terminal half of IQGAP1, encompassing GRD, RGCT, and CT, binds preferentially to the active, GTP-bound form of CDC42 (22–25) (see also Table 1). IQGAP1 GRD, which is structurally a RASGAP homologue but functionally an inactive RASGAP (26), has very recently been demonstrated to undergo interaction with CDC42 although with a substantially lower affinity than the larger protein fragment, containing GRD, RGCT, and CT (25, 26). These works together with homology modeling using RHOA-RHOGAP (27), CDC42-RHOGAP (28), and HRAS-RASGAP (29) complex structures provided structural models of IQGAP1 GRD that contacts the switch regions of active CDC42 (25, 26, 30). Remarkably, this model has been very recently confirmed by a dimeric GRD structure in complex with four molecules of the constitutive active CDC42^Q61L^ variant (31). Interestingly, RGCT contains two serines, 1441 and 1443, that have been shown to be phosphorylation sites by protein kinase Cϵ (PKCϵ) (32). Phosphomimetic mutations at these sites have been shown to significantly impair IQGAP1 interaction with CDC42 (24). This strongly indicates that regions or domains adjacent to the GRD are also critical for the interaction with the RHO family proteins.

To shed more light on a structure-function relationship of IQGAP1 interaction with CDC42 and RAC1, we purified different IQGAP1 variants (Fig. 1 and Table 1) and comprehensively investigated these interactions using both a time-resolved fluorescence stopped-flow spectrometry and fluorescence polarization under equilibrium condition using two different fluorescent reporters. The data provide unprecedented and novel mechanistic insights into the binding mode of IQGAP1 to RHO proteins and suggest a central role of the RGCT for IQGAP1 binding to the CDC42 and RAC1.

## Results

### 

#### 

##### Kinetics of the IQGAP1 Interaction with CDC42 and RAC1

**FIGURE 2.**
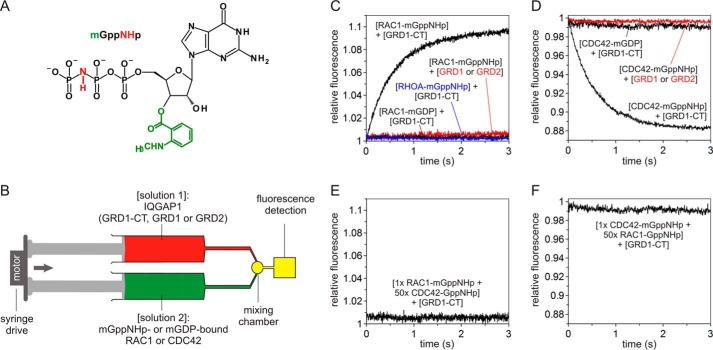
**GRD1-CT but not GRD selectively associates only with mGppNHp-bound, active RAC1 and CDC42.** *A*, chemical structure of mGppNHp, a fluorescently labeled, non-hydrolyzable GTP analog, used in this study. *B*, the stopped-flow device. The stopped-flow device consists of two motorized, thermostated syringes, a mixing chamber, and a fluorescence detector. Two different protein solutions indicated in *brackets* were rapidly mixed and transferred to a fluorescence detection cell within <4 ms. mGppNHp- or mGDP-bound RHO proteins were used in this study as the fluorescent reporter groups. *C* and *D*, association of GRD1-CT with active, mGppNHp-bound CDC42 and RAC1. Kinetics of association were followed by rapidly mixing 2 μm GRD1-CT, GRD1, or GRD2 with 0.2 μm mGppNHp- or mGDP-bound RAC1 (*C*) or CDC42 (*D*). The obtained data are the average of four to six independent measurements. The *k*_obs_ values obtained for the association of GRD1-CT with mGppNHp-bound CDC42 and RAC1 were 1.68 and 1.80 s^−1^, respectively. No change in fluorescence was observed for GRD1-CT- and mGDP-bound RAC1 or CDC42 (*black*), GRD1 or GRD2 with mGppNHp-bound RAC1 or CDC42 (*red*), and GRD1-CT with RHOA-mGppNHp (*blue*). *E* and *F*, overlapping binding sites of CDC42 and RAC1 for GRD1-CT. Association of RAC1-mGppNHp (0.2 μm) with GRD1-CT (2 μm) was blocked in the presence of excess amount of non-fluorescent CDC42-GppNHp (10 μm) (*E*). Association of CDC42-mGppNHp (0.2 μm) with GRD1-CT (2 μm) was blocked in the presence of excess amount of non-fluorescent RAC1-GppNHp (10 μm) (*F*).

Previous work has established that RHO proteins in complex with methylanthraniloyl (mant or m)-conjugated GppNHp (mGppNHp), a fluorescent and non-hydrolysable GTP analog (Fig. 2*A*), shows a change in fluorescence upon association with their downstream effectors using stopped-flow fluorometry (10). This signal can be used to monitor the kinetics of the effector interactions with RHO proteins. Thus, using stopped-flow fluorometry (Fig. 2*B*), we examined whether such a signal can be generated upon the interaction of IQGAP1 with RHO proteins (see “Experimental Procedures”). We observed a rapid change in fluorescence after mixing the GRD1-CT (comprising the C-terminal half of IQGAP1; Fig. 1) with mGppNHp-bound RAC1 (Fig. 2*C*) or CDC42 (Fig. 2*D*), which is directly related to the association reaction (see below). There was no change in fluorescence when RHOA-mGppNHp was mixed with GRD1-CT (Fig. 2*C*), clearly showing that RHOA, in contrast to CDC42 and RAC1, does not associate directly with IQGAP1 under these experimental conditions.

Interestingly, GRD1-CT binding to RAC1-mGppNHp resulted in an increase in fluorescence (Fig. 2*C*), whereas association with CDC42-mGppNHp led to a fluorescence decay (Fig. 2*D*). This indicates that CDC42 and RAC1, despite their high sequence identity (71%), obviously differ in regard to their binding modes with IQGAP1. Consistent with our results Owen *et al.* (25) have studied GRD1-CT interaction with a large panel of CDC42 and RAC1 variants and have suggested that CDC42 and RAC1 appear to have partially overlapping binding sites for IQGAP1 and may use different structural determinants to achieve high affinity binding. To examine this issue we performed competition experiments by mixing GRD1-CT with fluorescent RAC1-mGppNHp and an excess of non-fluorescent CDC42-GppNHp under otherwise the same conditions as above. Fig. 2*E* shows that the presence of CDC42-GppNHp completely blocked GRD1-CT association with RAC1-mGppNHp. A reverse experiment, mixing GRD1-CT with fluorescent CDC42-mGppNHp and an excess of non-fluorescent RAC1-GppNHp, led to the same results (Fig. 2*F*). These data indicated that CDC42 and RAC1, despite obvious differences, share an overlapping binding region for GRD1-CT.

Control experiments using mGDP-bound, inactive CDC42 and RAC1 proteins showed no change in fluorescence when they were mixed with GRD1-CT (Fig. 2, *C* and *D*). This result supports previous observations (30, 33, 34) and indicates that IQGAP1 primarily recognizes the switch regions of CDC42 and RAC1 by preferentially binding to their GTP-bound, active state. To investigate the IQGAP1-RHO protein interaction characteristics in more details, we purified two different GRD fragments designated as GRD1 and GRD2 (Fig. 1) and analyzed their association with mGppNHp-bound RAC1 or CDC42. In contrast to the GRD1-CT, none of these fragments remarkably show any change in the fluorescence signal (Fig. 2, *C* and *D*), even the highest actual concentration used for GRD1 and GRD2 (data not shown). One possible reason may be the small-sized “mant” as fluorescence reporter group (Fig. 2*A*). To examine this, we repeated the experiments under the same conditions by using tetramethylrhodamine (also called “tamra”), a different and much larger fluorescent reporter conjugated with GppNHp (tGppNHp; supplemental Fig. S2*A*). Data obtained with CDC42-tGppNHp clearly showed no GRD2 association signal (supplemental Fig. S2*B*). This result was unexpected because GRD is generally accepted as the RAC1 and CDC42 binding domain of IQGAP1 (24–26, 30). Our data suggest that GRD either does not directly interact with CDC42 and RAC1 or it associates outside the switch regions in a distant site from the fluorescent mant and tamra moieties of the nucleotide, thereby generating no change in the fluorescence signal.

##### IQGAP1 Possesses at Least Two CDC42 Binding Domains

**FIGURE 3.**
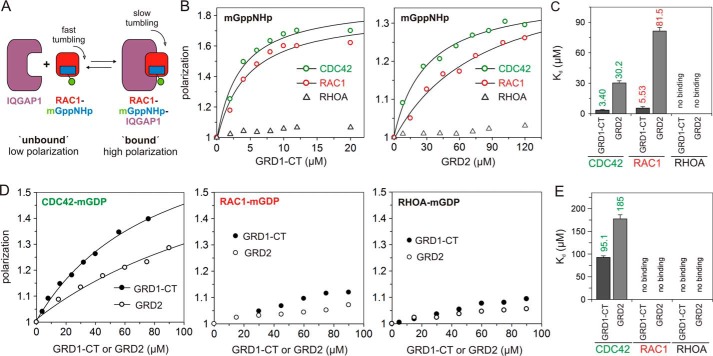
**IQGAP1 GRD binds CDC42 but not RAC1 in a nucleotide-independent manner.** *A*, fluorescence polarization assay. Fluorescence polarization signal of a fast tumbling fluorescent molecule, *e.g.* RAC1-mGppNHp in its unbound state, increased if a larger protein, *e.g.* IQGAP1, bound to it and formed a slow tumbling complex. *B*, fluorescence polarization experiments were conducted by titrating mGppNHp-bound, active forms of CDC42, RAC1, and RHOA (1 μm, respectively) with increasing concentrations of GRD1-CT (0–20 μm) or GRD2 (0–120 μm), respectively. *C*, evaluated dissociation constant (*K_d_*) shown as *bars* illustrates a significant difference in the binding properties of these two IQGAP1 proteins measured in *B. D*, fluorescence polarization experiments were conducted under the same conditions as in *B* using mGDP-bound, inactive forms of CDC42, RAC1, and RHOA. *E*, calculated *K_d_* values shown as *bars* clearly indicated interaction of GRD1-CT and GRD2 with CDC42-mGDP but not with RAC1 and RHOA. Data are expressed as the mean ± S.D. All experiments were performed in duplicate.

The next question addressed was whether IQGAP1 GRD can physically associate with parts of CDC42 and RAC1 other than their switch regions. We applied fluorescence polarization, a different approach to determine the binding affinity of the two proteins at equilibrium (Fig. 3*A*), and measured the dissociation constant (*K_d_*) of their interaction (35). Fig. 3, *B* and *C*, show that titration of mGppNHp-bound CDC42 and RAC1 with increasing amounts of not only GRD1-CT but, remarkably, also GRD2 led to an increase of polarization. These IQGAP1 domains, however, did not exhibit any association with RHOA-mGppNHp (Fig. 3, *B* and *C*). *K_d_* values obtained from these measurements showed that GRD1-CT is a high affinity binder as compared with GRD2, which shows 9- and 15-fold lower affinity for mGppNHp-bound CDC42 and RAC1, respectively (Fig. 3*C*; Table 1).

**TABLE 1:** **Data summary for the interaction of RHO proteins with IQGAP1 variants** The binding affinity of the IQGAP1 proteins for various RHO proteins has been analyzed in different ways including the following: SFF, stopped-flow fluorometry; FA, fluorescence assay; FP, fluorescence polarization; ITC, isothermal titration calorimetry; PRA, phosphate-release assay; SPA, scintillation proximity assay; SPR, surface plasmon resonance. n.s.o., no (fluorescence) signal observed; n.b.o., no binding observed.

IQGAP1 proteins	*K_d_**^a^*	Method	Reference
	μ*m*		
GRD1/RAC1-mGppNHp	n.s.o.	SFF	This study
GRD1/CDC42-mGppNHp	n.s.o.	SFF	This study
GRD2/RAC1-mGppNHp	n.s.o.	SFF	This study
GRD2/CDC42-mGppNHp	n.s.o.	SFF	This study
GRD2/CDC42-tGppNHp	n.s.o.	SFF	This study
GRD2/CDC42^Q61L^-tGppNHp	n.s.o.	SFF	This study
GRD1-CT/RAC1-mGppNHp	0.94	SFF	This study
GRD1-CT/RAC1-mGDP	n.s.o.	SFF	This study
GRD1-CT/CDC42-mGppNHp	0.30	SFF	This study
GRD1-CT/CDC42-tGppNHp	0.38	SFF	This study
GRD1-CT/CDC42^Q61L^-tGppNHp	0.05	SFF	This study
GRD1-CT/CDC42-mGDP	n.s.o.	SFF	This study
GRD1-CT/RHOA-mGppNHp	n.s.o.	SFF	This study
GRD1-RGCT^WT^/RAC1-mGppNHp	2.81	SFF	This study
GRD1-RGCT^WT^/CDC42-mGppNHp	2.72	SFF	This study
GRD1-RGCT^WT^/CDC42-tGppNHp	1.45	SFF	This study
GRD1-RGCT^S1441E^/RAC1-mGppNHp	3.21	SFF	This study
GRD1-RGCT^S1441E^/CDC42-mGppNHp	2.05	SFF	This study
GRD1-RGCT^S1443E^/RAC1-mGppNHp	6.26	SFF	This study
GRD1-RGCT^S1443E^/CDC42-mGppNHp	7.03	SFF	This study
GRD1-RGCT^S1443E^/CDC42-tGppNHp	4.19	SFF	This study
CT/RAC1-mGppNHp	n.s.o.	SFF	This study
CT/CDC42-mGppNHp	n.s.o.	SFF	This study
GRD1-RGCT/CDC42-GDP	1.30	SPR	(24)
GRD1-RGCT^S1443D^/CDC42-GDP	0.81	SPR	(24)
GRD1-RGCT^S1441E^/CDC42-GDP	220.0	SPR	(24)

IQGAP1 proteins	e *K_d_**^b^*	Method	Reference
	μ*m*		
GRD2/RAC1-mGppNHp	81.50	FP	This study
GRD2/RAC1-mGDP	n.b.o	FP	This study
GRD2/CDC42-mGDP	185	FP	This study
GRD2/CDC42-mGppNHp	30.20	FP	This study
GRD2/CDC42^Q61L^-mGppNHp	2.37	FP	This study
GRD2/CDC42^Q61L^-tGppNHp	1.13	FP	This study
GRD2/RHOA-mGppNHp	n.b.o.	FP	This study
GRD1-CT/RAC1-mGppNHp	5.53	FP	This study
GRD1-CT/CDC42-mGDP	95.10	FP	This study
GRD1-CT/CDC42-mGppNHp	3.40	FP	This study
GRD1-CT/CDC42^Q61L^-mGppNHp	0.83	FP	This study
GRD1-CT/CDC42^Q61L^-tGppNHp	0.52	FP	This study
GRD1-CT/RHOA-mGppNHp	n.b.o.	FP	This study
GRD1-RGCT^WT^/RAC1-mGppNHp	4.60	FP	This study
GRD1-RGCT^S1441E^/RAC1-mGppNHp	6.70	FP	This study
GRD1-RGCT^S1443E^/RAC1-mGppNHp	26.70	FP	This study
GRD1-RGCT^WT^/CDC42-mGppNHp	4.60	FP	This study
GRD1-RGCT^S1441E^/CDC42-mGppNHp	3.60	FP	This study
GRD1-RGCT^S1443E^/CDC42-mGppNHp	16.30	FP	This study
GRD1-CT/CDC42-mdGTP	0.028	FA	(47)
GRD1-CT/RAC1^Q61L^-[^3^H]GTP	0.018	SPA	(25)
GRD2*^c^*/RAC1^Q61L^-[^3^H]GTP	n.b.o.	SPA	(25)
GRD1-CT/CDC42^Q61L^-[^3^H]GTP	0.024	SPA	(25)
GRD2*^c^*/CDC42^Q61L^-[^3^H]GTP	0.14	SPA	(25)
GRD2/CDC42^Q61L^-GTP	1.30	ITC	(26)
GRD2/CDC42^Q61L^	0.1	ITC	(31)
GRD2/CDC42-GDP	n.b.o.	ITC	(26)

IQGAP1 proteins	*K_ic_*	Method	Reference
	μ*m*		
GRD1-CT/CDC42^Q61L^-GTP	0.082	PRA	(76)
GRD1-CT/CDC42-GTP	0.39	PRA	(23)
GRD1-CT/RAC1-GTP	2.13	PRA	(23)

*^a^* Kinetic conditions provide individual association and dissociation rate constants (*k*_on_ and *k*_off_) and determine the dissociation constants (*K_d_*).

*^b^* Equilibrium conditions determine the equilibrium dissociation constants (e*K_d_*).

*^c^* Encompasses amino acids 950–1407.

*^d^* Competitive reaction conditions; for example, inhibition of the intrinsic GTP hydrolysis reaction of the RHO proteins, which determines the equilibrium inhibition constant (*K_i_*).

The explanation for our observations regarding interaction of GRD with the active form of RAC1 is simple; in direct mode, only a change in fluorescence can be observed when the associating protein (*e.g.* GRD1-CT) binds in close vicinity of the fluorophore (mant moiety of the bound mGppNHp or tamra moiety of the bound tGppNHp) on the surface of CDC42 and RAC1 (Figs. 2 and supplemental Fig. S2). This surface area of RHO proteins covers the switch regions that change their conformation upon a GDP/GTP exchange (2). This is of fundamental importance because effectors (such as IQGAP1) first bind to the switch regions, which represent the recognition sites. Binding of other additional effector domains determines the specificity of the interaction, which is ultimately required for effector activation (2, 10, 36). So far our data suggest that the binding of the GRD domain to RHO proteins seems to happen outside the switch regions and, thus, would be expected to be independent of the nucleotide status of the RHO protein. To prove this idea, we repeated the measurements by using mGDP-bound, inactive RHO proteins. Both GRD1-CT and GRD2 were able to interact with mGDP-bound CDC42, although with very low affinities but, surprisingly, not with RAC1 (Fig. 3, *D* and *E*; Table 1); the latter was also the case for RHOA. This strongly suggests that the molecular nature of IQGAP1 interaction with CDC42 partially differs from that with RAC1, particularly with regard to the role of GRD. We propose that IQGAP1 harbors at least two distinct binding domains. RGCT contributes to a high affinity binding to the switch regions of the GTP-bound, active CDC42 and RAC1. GRD more selectively recognizes active forms of CDC42 and RAC1 but also binds to other regions outside of the switch regions of CDC42, obviously in a nucleotide-independent manner.

##### RGCT Is Primarily Critical for the IQGAP1 Association with CDC42 and RAC1

**FIGURE 4.**
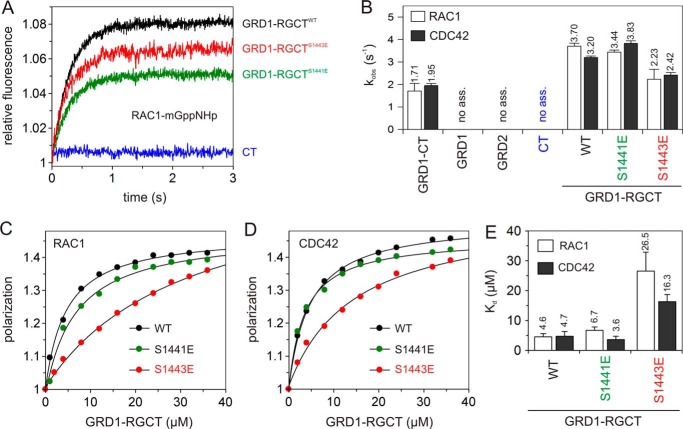
**IQGAP1 variants significantly differ in their interaction properties with CDC42 and RAC1.** *A* and *B*, association of different GRD1-RGCT variants and CT (2 μm, respectively) with mGppNHp-RAC1/CDC42 (0.2 μm) was measured. *A*, association of GRD1-RGCT^WT^ (*black*), GRD1-RGCT^S1441E^ (*green*), and GRD1-RGCT^S1443E^ (*red*), but not with CT (*blue*), with RAC1-mGppNHp. *B*, the *k*_obs_ values, shown as *bars*, comparatively illustrate association rates of GRD1-CT, GRD1-RGCT^WT^, and GRD1-RGCT^S1441E^ with mGppNHp-bound forms of CDC42 and RAC1, which is significantly reduced in the case of GRD1-RGCT^S1443E^ and completely absent in the case of GRD1, GRD2, and CT under these experimental conditions. *C–E*, fluorescence polarization experiments were conducted to measure the interaction of mGppNHp-bound forms of RAC1 (*C*) and CDC42 (*D*) with increasing concentrations of GRD1-RGCT variants (WT, S1441E, and S1443E; 0–36 μm, respectively). *E*, calculated *K_d_* values, shown as *bars*, reveal a significant decrease in the binding affinities of GRD1-RGCT^S1443E^ as compared with GRD1-RGCT^WT^ and GRD1-RGCT^S1441E^. Data are expressed as the mean ± S.D. All *k*_obs_ measurements were performed in triplicate, and fluorescence polarization experiments were conducted in duplicate.

To further prove the critical role of the C-terminal domains of IQGAP1 beyond GRD, we generated various deletion and point mutations of IQGAP1 (Fig. 1). We measured the effect of the last 99 amino acids of IQGAP1 on CDC42 and RAC1 binding by using purified CT and GRD1-RGCT (IQGAP1^877–1558^), which lacks this region (Fig. 1). In contrast to GRD1-RGCT, isolated CT itself did not show, similarly to GRD1 and GRD2, any change in fluorescence upon mixing it with CDC42 and RAC1, respectively (Figs. 2, *C* and *D*, and 4*A*). For comparison, the *k*_obs_ values were obtained by fitting all the data as single exponentials and are plotted as bar charts, which illustrates that interaction of GRD1-RGCT with CDC42 and RAC1 has different *k*_obs_ compare with GRD1-CT (Fig. 4*B*). These data emphasize the essential role of RGCT for direct association of IQGAP1 with the switch regions of CDC42 and RAC1. We were able to purify RGCT (aa 1276–1575) and also GST-fused RGCT-CT (aa 1276–1657) (Fig. 1). Both proteins, however, tend to strongly oligomerize and elute in void volume (supplemental Fig. S1*B*) such that we were unable to test binding to CDC42 and RAC1 (data not shown).

Interestingly, RGCT contains two PKCϵ phosphorylation sites (Ser-1441 and Ser-1443; Fig. 1) (24, 32), which differently affect GRD1-RGCT association with RAC1-mGppNHp upon their phosphomimetic mutations. To investigate the effect of IQGAP1 phosphorylation on the interaction with RAC1 and CDC42 in more detail, we generated and purified phosphomimetic mutants of the GRD1-RGCT^WT^ domain (GRD1-RGCT^S1441E^ and GRD1-RGCT^S1443E^).

In contrast to GRD1-RGCT^WT^ and GRD1-RGCT^S1441E^, with Ser-1441 substituted by Glu, GRD1-RGCT^S1443E^ with Ser-1443 substituted by Glu impaired the observed association of GRD1-RGCT with active CDC42 and RAC1 (Fig. 4, *A* and *B*). Determination of the *K_d_* values for the RAC1 and CDC42 binding GRD1-RGCT variants using fluorescence polarization showed that GRD1-RGCT and GRD1-RGCT^S1441E^ exhibited binding affinities for RAC1 and CDC42 in a similar range, which significantly differs from GRD1-RGCT^S1443E^ (Fig. 4, *C–E*; Table 1). These data suggest that RGCT is a critical domain in IQGAP1 that is primarily responsible for the recognition of switch regions and association with the GTP-bound CDC42 and RAC1. In addition, Ser-1443 phosphorylation appears to serve as a regulatory switch for these interactions. Our data are in line with the study of Elliott *et al.* (24) and support the previous study by Li *et al.* (32), which highlighted the importance of this phosphorylation event in IQGAP1-mediated cellular signaling.

To prove that these IQGAP1 variants, including GRD1-RGCT^S1443E^, are structurally intact, we conducted circular dichroism (CD) and analytical size exclusion chromatography (SEC) analyses, respectively (supplemental Fig. S1). The CD measurements demonstrate that all IQGAP1 variants used in this study were well structured and did not reveal any significant differences in the secondary structure between them. In addition, SEC data revealed that the IQGAP1 variants are properly folded and exist as dimeric proteins as seen for GRD1-RGCT and GRD2 (supplemental Fig. S1). The latter is in agreement with the dimeric GRD structure in complex with CDC42^Q61L^ (31).

##### Modulatory Effects of CT on IQGAP1 Interaction with CDC42 and RAC1

**FIGURE 5.**
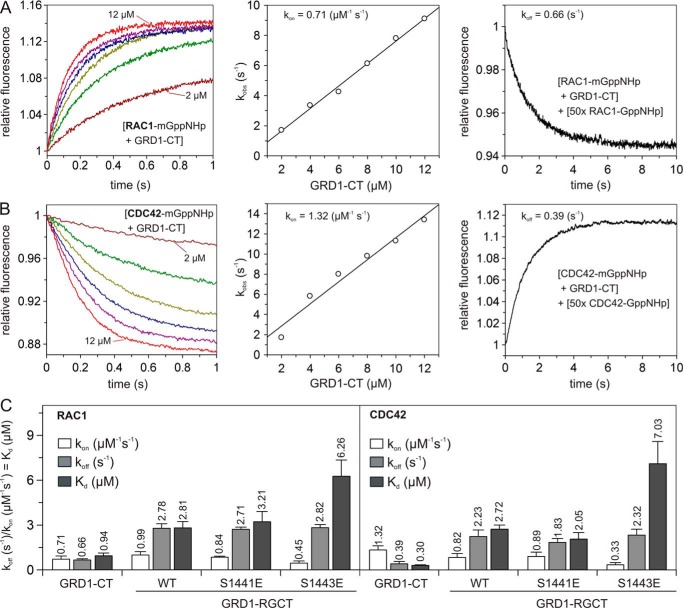
**Phosphomimetic mutation of RGCT and deletion of CT affect the IQGAP1 association with CDC42 and RAC1.** *A* and *B*, individual rate constants for the GRD1-CT interaction with RAC1 and CDC42 are represented in *A* and *B. Left panel*, association of mGppNHp-bound RAC1 or CDC42 (0.2 μm, respectively) with increasing concentrations of GRD1-CT (2 to 12 μm). *Middle panel*, evaluated association rate constant (*k*_on_) from the plot of the *k*_obs_ values, obtained from the exponential fits to the association data in the left panel against the corresponding concentrations of the GRD1-CT. *Right panel*, evaluated dissociation rate constant (*k*_off_) measured by the displacement of the GRD1-CT (2 μm) from its complex with mGppNHp-bound RAC1 or CDC42 (0.2 μm, respectively) in the presence of excess amounts of non-fluorescent RAC1-GppNHp (10 μm). Other kinetics are given in supplemental Figs. S1 and S2. *C*, calculated individual rate constants for the interaction of the IQGAP1 variants with RAC1 and CDC42, respectively, plotted as *bar charts*. Dissociation constants (*K_d_*) were obtained from the ratio *k*_off_/*k*_on_. Data are expressed as the mean ± S.D. All *k*_obs_ measurements experiments were accomplished in triplicate.

To understand the IQGAP1 interaction with CDC42 and RAC1 in more details, kinetic measurements were performed. This allowed us to obtain individual rate constants *k*_on_ and *k*_off_ for the rate of association and dissociation, respectively, as well as the dissociation constant (*K_d_*) calculated from the ratio of *k*_off_/*k*_on_. To calculate the *k*_on_ value, association of mGppNHp-bound RAC1 and CDC42 with increasing concentrations of GRD1-CT was measured, leading to changes in fluorescence curves characterized by individual *k*_obs_ values (Fig. 5, *A* and *B*, *left panels*). Obtained *k*_obs_ values were fitted in a linear fashion as a function of GRD1-CT concentrations, and the slope of the fitted line provided the *k*_on_ (Fig. 5, *A* and *B*, *middle panels*). Displacement of GRD1-CT from fluorescently labeled RAC1 and CDC42 was measured by mixing the RAC1/CDC42-mGppNHp-GRD1-CT complex with an excess amount of non-fluorescent RAC1/CDC42-GppNHp. Fig. 5, *A* and *B*, *right panels*, shows a mono-exponential fluorescence decay for RAC1 and increase for CDC42 yielding the *k*_off_ value for RAC1 and CDC42, respectively. From the ratio of *k*_off_/*k*_on_, we calculated the dissociation constant (*K_d_*) of 0.94 and 0.30 μm for RAC1 and CDC42, respectively, which is comparable with the values obtained by fluorescence polarization in this study and other methods in other laboratories (Table 1; Fig. 5*C*) (23, 25, 26). To further confirm our obtained results with mGppNHp, CDC42 was loaded with larger fluorescent nucleotide tGppNHp (supplemental Fig. S2 *A* and Fig. 2*A*). Kinetic data obtained for the interaction of CDC42-tGppNHp with GRD1-CT, GRD1-RGCT, and GRD1-RGCT^S1443E^ were very similar to the data described above for CDC42-mGppNHp (supplemental Fig. S2 and Fig. 5*A*).

CDC42 as compared with RAC1 exhibited a faster *k*_on_, a slower *k*_off_, and therefore, a 3-fold higher binding affinity for GRD1-CT (Fig. 5, *B* and *C*). GRD1-RGCT, lacking the C-terminal CT domain, strikingly revealed a 4–6-fold faster *k*_off_ in comparison to GRD1-CT, whereas the *k*_on_ values remained largely the same (Figs. 5*C*, supplemental Figs. S3 and S4). Thus, C-terminal truncation of IQGAP1 reduced its overall affinity for CDC42 and RAC1, indicating that CT either acts directly as an additional CDC42 and RAC1 binding domain or may indirectly stabilize RGCT and/or GRD binding to CDC42 and RAC1.

To examine a direct interaction of IQGAP1 CT, encompassing the C-terminal 99 amino acids (Fig. 1), we used the RHO proteins and isolated the CT domain as a GST fusion protein and measured their interaction using fluorescence polarization. The data revealed that CTs have a binding capacity of its own for CDC42, RAC1, and RHOA that is rather very low (supplemental Fig. S5). In addition to RGCT and GRD, CT may play, as a very low affinity binder, a direct role in the CDC42 and RAC1 interaction. CT also seems to bind RHOA, which probably means that its binding region on the surface of these prominent members of the RHO family must be conserved (see “Discussion”).

From the two reported phosphorylation sites (32), Ser-1443 turned out to be critical for the IQGAP1 interaction with CDC42 and RAC1 but not Ser-1441 (Fig. 4). Kinetic data shown in Fig. 5*C*, supplemental Figs. S3 and S4 clearly revealed that Ser-1443 substitution for Glu more strongly affected the GRD1-RGCT association with CDC42 and also RAC1 as compared with the dissociation reaction. The rate constants obtained for GRD1-RGCT^S1441E^ were as the wild-type (WT) (Fig. 5*C*). Thus, we propose that RGCT is critical for IQGAP1 association with CDC42 and RAC1, which is modulated by Ser-1443 phosphorylation, whereas GRD and probably CT may control the resident time and the off-rate of the protein complex.

##### Q61L Mutation Generates a Very High Affinity of CDC42 for IQGAP1

**FIGURE 6.**
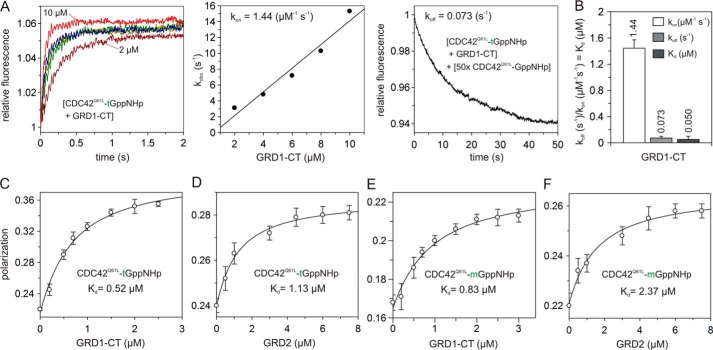
**Q61L mutation greatly increased CDC42 affinity for IQGAP1.** *A*, individual rate constants for the GRD1-CT interaction with CDC42^Q61L^. Association of tGppNHp-bound CDC42^Q61L^ (0.2 μm) with increasing concentrations of GRD1-CT (2 to 10 μm) is shown in the *left panel*. The *middle panel* shows evaluated association rate constant (*k*_on_), and the *right panel* shows the evaluated dissociation rate constant (*k*_off_) measured by the displacement of the GRD1-CT (2 μm) from its complex with tGppNHp-bound CDC42 (0.2 μm) in the presence of excess amounts of non-fluorescent CDC42^Q61L^-GppNHp (10 μm). *B*, calculated individual rate constants for the interaction of the GRD1-CT with CDC42^Q61L^, plotted as *bar charts*. A dissociation constant (*K_d_*) of 0.05 μm was obtained from the ratio *k*_off_/*k*_on_. *C–F*, fluorescence polarization were performed by titrating CDC42^Q61L^-tGppNHp (1 μm) with increasing concentrations of GRD1-CT (0–2.5 μm) (*C*) and GRD2 (0–7.5 μm) (*D*) or CDC42^Q61L^-mGppNHp with increasing concentrations of GRD1-CT (0–3 μm) (*E*) and GRD2 (0–7.5 μm) (*F*).

Two different laboratories reported a high affinity for the interaction of GRD with CDC42 (25, 31), which clearly contrasts with our data on very low affinity binding (Fig. 3; Table 1). An obvious reason for this discrepancy is that Owen *et al.* (25) and LeCour *et al.* (31) use a constitutive active variant of CDC42, CDC42^Q61L^, and we used CDC42^WT^. Contrary to these groups, we did not observe any significant interaction of RAC1 with GRD, and the *K_d_* values we obtained for CDC42 were up to 300-fold lower (Table 1). To evaluate this discrepancy, we generated and purified CDC42^Q61L^ and measured its interaction with GRD1-CT and GRD2 using both stopped-flow and fluorescence polarization. Similar to CDC42^WT^, no association was observed for GRD2 and CDC42^Q61L^ (data not shown), but kinetic measurements revealed a 7-fold stronger interaction of GRD1-CT with CDC42^Q61L^ with a *K_d_* of 50 nm (Fig. 6, *A* and *B*; Table 1). Equilibrium measurement using fluorescence polarization exhibited a much stronger interaction of GRD1-CT and GRD2 with CDC42^Q61L^ as compared with CDC42^WT^ (Fig. 6, *C–F*; Table 1). Our analyses clearly demonstrate that substitution of Gln-61 by Leu engaged binding of effector domains. An analysis of the GRD-CDC42^Q61L^ structure (31) showed that Leu-61 is indeed in direct contact with GRD in all four interacting interfaces (data not shown). Collectively, we recapitulate that CDC42^Q61L^ is not a perfect analog of CDC42^WT^-GTP especially in the study of the downstream effectors.

## Discussion

Association of downstream effectors with their specific small GTPases has been generally accepted to release them from an autoinhibited state, resulting in their activation. The switch regions of small GTPases have been previously proposed as the first binding site for the downstream effectors. When this first contact is achieved, then additional contacts outside the switch regions are required to achieve effector activation (2). The molecular mechanism of this process, which is critical for signal transduction, is unclear for most effectors, especially for scaffolding proteins. A straight-forward example is WASP, which is able to activate the actin-related proteins ARP2/3 if its C-terminal ARP2/3-binding domains are unmasked upon association with CDC42 (10). This process controls assembly of actin filaments (37). In contrast, IQGAP1 is a multifaceted protein that employs different domains in an assembly of multitude protein complexes, coordinating different processes in a variety of cell types (14–17).

A key role has been previously assigned to the IQGAP1 GRD association with the switch regions of CDC42 and RAC1 (25, 26), and this has been generally accepted as an interaction model for these proteins, albeit with suggestions that critical determinants for RAC1 binding to IQGAP1 lie outside this region (25). Very recently, the crystal structure of GRD-CDC42^Q61L^ complex has been published (31). It consists of four CDC42^Q61L^ molecules binding to a dimeric GRD. An inspection of this structure revealed that all four contact sites engage the switch regions of CDC42^Q61L^ (data not shown). However, our kinetic and equilibrium measurements conducted with various deletion and point mutations of IQGAP1, and the use of two different fluorescence reporter groups clearly exclude a central role for GRD in the recognition of RHO proteins via their switch regions. Thus, we propose a binding of GRD adjacent to or outside the switch regions of CDC42. Instead, a region next to GRD appeared as the central domain for the association of IQGAP1 with the switch regions of both CDC42 and RAC1. Mimetic mutations of the PKCϵ phosphorylation site Ser-1443 in this domain significantly affected IQGAP1 association with CDC42 and RAC1. In contrast to CDC42 and RAC1 and unlike previous cell-based studies (38, 39) but very much in line with early studies (33, 40–45), no physical interaction of neither GRD2 or GRD1-CT was observed with RHOA using both kinetic and equilibrium measurements (Figs. 2*C* and 3, *B* and *C*). This clearly indicates that the observed IQGAP1 association with RHOA is indirectly mediated by other proteins in co-immunoprecipitates. Interaction of GRD1-CT with CDC42 and RAC1 is strong and GTP-dependent. GRD made a considerable contribution to an overall binding affinity of IQGAP1 to CDC42 and RAC1, although its role in these interactions turned out to be rather different (Fig. 3). GRD underwent a very weak interaction with mGDP-bound, inactive CDC42 but not with RAC1-mGDP. This rather suggests that GRD binds to a surface outside the switch regions of CDC42, which is obviously not conserved in RAC1. Furthermore, our data also demonstrate that the extreme C-terminal region of IQGAP1, CT (99 aa), may also bind, although with a very low affinity, to a common site of CDC42 and RAC1 that is also shared by RHOA (supplemental Fig. S5).

Previous studies by other groups have shown that shorter IQGAP1 fragments, encompassing the GRD domain, are responsible for the CDC42 and RAC1 interactions. Zhang and coworkers showed that an activated form of CDC42 is able to bind IQGAP1 GRD1-CT (aa 864–1657) (76). One year later the same group reported that not only CDC42 but also RAC1, although with lower affinity, could interact to GRD1-CT (23). Afterward, Nomanbhoy and Cerione (47) have shown that GRD1-CT interacts tightly with CDC42-mGTP using a fluorescence assay. *Owen* et al. (25) have also reported that a GRD protein (aa 950–1407) was able to tightly bind CDC42^Q61L^ with a *K_d_* value of 140 nm but failed to bind RAC1(Q61L) using a scintillation proximity assay. In contrast, GRD1-CT has shown a much higher affinity for the Q61L mutant of not only CDC42 but also of RAC1, and yet the GRD was proposed to be the binding domain of IQGAP1 that associates with the switch regions of CDC42. Correspondingly, Kurella *et al.* (26) reported that GRD2 (aa 962–1345) binds CDC42^Q61L^ in a GTP-dependent manner with an affinity of 1.3 μm using isothermal titration calorimetry. These biochemical data (summarized in Table 1) along with the homology modeling based on the RAS-RASGAP structure (29) provided a first structural model of IQGAP1 GRD contacting the switch regions of the CDC42, which is generally accepted in the community (24–26, 30, 48). A very recent study from the same laboratory has determined the crystal structure of GRD with CDC42^Q61L^ (31), which is very similar to their previous model (26). On the contrary, we observed different properties of GRD1-CT and GRD2 in their interactions with CDC42^WT^. This was evidenced by kinetic measurements of GRD1-CT and GRD1-RGCT association, but not GRD, with CDC42 and RAC1 proteins (Figs. 2 and 4; no changes in fluorescence were observed with GRD). Similar data were obtained by using tamra, a different fluorescence reporter instead of mant. Equilibrium measurements using fluorescence polarization not only substantiated the essential role of IQGAP1 RGCT in a GTP-dependent interaction with CDC42 and RAC1, in agreement with our kinetic analysis, but also provided striking insights into the main feature of IQGAP1 GRD. Our quantitative analysis under equilibrium conditions clearly revealed that GRD undergoes a low-affinity interaction with CDC42, but its binding in contrast to RGCT is partially nucleotide-dependent.

mGDP-bound RHOA and particularly RAC1 did, however, not reveal any interaction with IQGAP1 GRD. A faster *k*_on_, a slower *k*_off_, and a lower *K_d_* of GRD1-CT for CDC42, in direct comparison to RAC1, strongly support our data from fluorescence polarization and demonstrated that not only RGCT but also GRD bind mGppNHp-bound CDC42 and RAC1 (Fig. 3). The significance of RGCT as a GTP-dependent interacting domain for CDC42 and RAC1 was proved using a single point mutant of GRD1-RGCT (Ser-1443 substituted by Glu), which led to the abolition of a GTP-dependent interaction of GRD1-RGCT, whereas nucleotide-independent association with CDC42 through GRD was unchanged. Ser-1443 was identified as the major site phosphorylated on IQGAP1 in intact cells treated with PMA (phorbol 12-myristate 13-acetate) (32). Phosphomimetic mutation of Ser-1441, a second phosphorylation site, that was shown to be phosphorylated to a lesser extent as compared with Ser-1443 (32), did not affect the interaction with either CDC42 or RAC1. Grohmanova *et al.* (33) previously showed via GST pulldown experiments and using MCF10A cell lysate that in the presence of phosphatase inhibitor there is a significant reduction in the interaction between IQGAP1 and CDC42-GTP in contrast to nucleotide-depleted CDC42, which binds to phosphorylated IQGAP1 much more strongly. In addition, our data have clearly demonstrated that the region upstream of GRD2 (aa 863–961) is dispensable for the CDC42 and RAC1 interaction.

Another interesting issue was a significantly faster dissociation of GRD1-RGCT (lacking the CT domain) from CDC42 and RAC1 as compared with GRD1-CT. This clearly indicates an involvement of the very C-terminal 99 amino acids (CT) in the overall binding affinity of GRD1-CT for CDC42 and RAC1 (Fig. 5*C*). Our fluorescence polarization measurements showed that isolated CT has the tendency to associate with CDC42, RAC1, and also RHOA although with low affinity (supplemental Fig. S5).

### 

#### 

##### A Multiple-step Binding Mechanism

Kinetic and equilibrium measurements conducted in this study challenge the paradigm that the ability of IQGAP1 to interact with RAC/CDC42 proteins is mainly attributed to its GRD. We propose that GRD1-CT, the C-terminal 795 amino acids of IQGAP1, encompasses at least 3 distinct domains that may differently interact with CDC42 and RAC1 at different contact sites in a multistep manner. The switch regions of the RHO family proteins are the first binding site for the downstream effectors. Once it is occupied, additional contacts outside the switch regions are required to guarantee effector activation (2, 10). Accordingly, RGCT appears as a central domain that rapidly associates with the switch regions of GTP-bound CDC42 and RAC1 but not RHOA. CT may additionally bind to a different site conserved in RAC1 and CDC42 and extend the residence time of the respective complexes. A possible interaction of CT with RHOA seems to be physiologically irrelevant simply because RGCT and GRD do not recognize RHOA, at least not under experimental conditions in this study. These interactions may induce a local conformational change enabling GRD to bind selectively to GTP-bound CDC42 and RAC1 but not RHOA. Such sequential but differential association of IQGAP1 with CDC42 *versus* RAC1 can be envisaged as conformational changes within IQGAP1, enabling a set of interactions at structurally accessible and available regions with its downstream targets depending on the upstream signals and the cell fate. The fact that GDP-bound CDC42, but not RAC1, is able to interact with GRD suggests that GRD may undergo a low affinity complex with GDP-bound, inactive CDC42 proteins outside or adjacent to its switch regions in a way that is independent of the upstream signals, providing GRD is structurally accessible and available for interactions. This may also be a model for the IQGAP1-mediated scaffolding of the CDC42-GTP-WASP complex regulating actin assembly, extension of lamellipodia, and promotion of dendritic spine head formation (15, 49, 50).

##### Concluding Remarks

Protein-protein interaction studies on the active, GTP-bound form of CDC42 and RAC1 has identified IQGAP1 as a downstream effector (22–26, 30, 34, 51–54). Accumulating evidence supports diverse roles for the IQGAP1 interaction with CDC42 and RAC1 in vertebrates, which has significance for a variety of biological functions. However, the nature of such protein-protein recognition processes has remained obscure. Initially it was thought that modulation of the cytoskeletal architecture is the primary function of the interaction of IQGAP1 with RHO proteins, but it is now clear that it has many critical physiological roles beyond the cytoskeleton. However, the consequences of different interactions of IQGAP1 with CDC42 *versus* RAC1 remained to be addressed.

Lack of GRD interaction with RAC1 may be compensated by calmodulin as an accessory protein, which has been reported as binding partner of RAC1 and IQGAP1 (55–57). The ability of GRD in binding outside the switch regions of CDC42 may facilitate the scaffolding function of IQGAP1 in localizing CDC42 and WASP at specific sites.

It is also of major interest to address the question of whether the C-terminal domains RGCT and CT compete in binding to CDC42 and RAC1 with other proteins, including CLIP-170, E-cadherin, β-catenin, adenomatous polyposis coli (APC), mDIA, CLASP2, TSG101, SEC3/8, and SMG9. These proteins have also been reported to associate with RGCT and CT (15, 41, 58–63). Adenomatous polyposis coli has been shown to activate ASEF, a CDC42-specific Dbl protein (64), at the leading edge of a migrating cell, which may thereby initiate CDC42-IQGAP1 signaling.

There is one obvious consequence of IQGAP1 as a RAC1/CDC42 effector; IQGAP1 not only has direct interactions with the small GTPases but also has been detected in a complex with an effector (PAK6) and a regulator (TIAM1) of small G proteins (65). Immunoprecipitation of IQGAP1 from human pulmonary artery endothelial cells isolated a complex containing TIAM1, RAC1, Src, cortactin, p47phox, and phospholipase D2 (65) but the direct interaction of TIAM1 and IQGAP1 has not been reported yet, and the possible biological relevance of the interaction remains unknown.

Subcellular localization of RHO GTPases to cellular membranes is known to be critical for their biological activity. This is achieved by a hypervariable region (HVR) (66) and a lipid anchor in their C-terminal tail at a distinct cysteine residue in the C*AAX* motif (C is cysteine, *A* is any aliphatic amino acid, and *X* is any amino acid) (67). RHOGDI is known to dislodge RHO proteins from the plasma membrane (68). As IQGAP1 also binds RHOGDI (20), it would be interesting to know whether IQGAP1 is a displacement factor for the RHOGDI complex with RAC1 or CDC42.

Another issue is the intramolecular autoregulation and autoinhibitory of IQGAP1 by its domains. Based on our structural modeling, we hypothesize that the calponin homology domain may act as an autoinhibitory domain that must be released in the course of IQGAP1 activation. We hypothesize that two proteins that may release IQGAP1 from its autoinhibited state may in fact be RAC1-GTP and calmodulin. The interaction of calmodulin with RAC1 (69) and IQGAP1 (45) has been reported, but the role of calmodulin in enhancing the RAC1-mediated IQGAP1 activation is not investigated yet. Another significant question to be answered is the possible role of these interactions in carcinogenesis. Published results suggest that blocking IQGAP1-CDC42 and IQGAP1-RAC1 complex formation will decrease the amount of active forms of CDC42 and RAC1 in carcinoma cells and thus reduce tumorigenesis. Therefore, antagonists that disrupt the binding of IQGAP1 to RAC1 and/or CDC42 could prevent tumor invasion, proliferation, and migration and could act as specific chemotherapeutic agents. However, the design of such antagonists absolutely and critically requires knowledge of the key protein-protein interfaces between IQGAP1 and these small GTPases. The data reported here provide an important step in defining these sites.

## Experimental Procedures

### 

#### 

##### Constructs

Coding sequences for RHO proteins and IQGAP1 variants were amplified using sequence-specific primer and cloned into the expression vector. Different pGEX vectors encoding an N-terminal glutathione *S*-transferase (GST) fusion protein were used for the overexpression ofvarious human genes: different IQGAP1 (accession number P46940) regions (aa 863–1345, 863–1657, 877–1558, 1276–1657, 1276–1575, 1576–1657) and also C-terminal truncated RAC1 (aa 1–179), CDC42 (aa 1–178), and RHOA (aa 1–181). pMCSG7 vector was used for overexpression of IQGAP1 aa 962–1345 and pET46 EkLIC vector (Merck) for the overexpression of IGQAP1 S1441E and S1143E mutants (aa 877–1558) as His-tagged proteins. The Kazusa cDNA clone KIAA0051 (70) was used as a template for site-directed mutagenesis.

##### Proteins

All proteins were purified according to the protocols described (3, 71, 72). Briefly, *Escherichia coli* strains pLysS BL21(DE3), CodonPlusRIL, or BL21(Rosetta) were transformed and used to purify the respective protein. Protein extraction was carried out by incubating cells at 4 °C with DNase I (10 μg ml^−1^) and lysozyme (10 μg ml^−1^) followed by cell lysis using a microfluidizer (model M110S, Microfluidics Corp.). Bacterial lysates were centrifuged to collect soluble fractions, and tagged proteins were isolated from the supernatant via Ni-NTA or GST affinity purification. If needed, the GST tag was cleaved with tobacco etch virus protease (4 units mg^−1^, 4 °C, overnight), and proteins were subjected to gel filtration using a standard buffer containing 30 mm Tris-HCl, pH 7.5, 150 mm NaCl, 5 mm MgCl_2_, and 3 mm DTT. All purified proteins were analyzed by SDS-PAGE (Fig. 1) and stored as either tag-fused or cleaved protein at −80 °C.

Nucleotide-free RHO proteins were prepared using alkaline phosphatase (Roche Applied Science) and phosphodiesterase (Sigma) at 4 °C as described (73). Various fluorescence reporter groups, including mant and tamra, have been coupled to 2′ (3′)-hydroxyl group of the ribose moiety of GDP and GppNHp via ethylenediamine (EDA) to obtain fluorescent nucleotide (Jena Bioscience).

##### Stopped-flow Fluorescence Measurements

Kinetics measurements were monitored by stopped-flow apparatus (Hi-Tech Scientific SF-61 with a mercury xenon light source and TgK Scientific Kinetic Studio software) and performed as described (72). Equal volumes of two different solutions containing IQGAP1 (different concentrations) or RHO protein (0.2 μm) were rapidly shot into a mixing chamber, and the fluorescence was directly detected after a dead time of <4 ms (Fig. 2*B*). Buffer contained 30 mm Tris-HCl, pH 7.5, 10 mm KH_2_PO_4_/K_2_HPO_4_, 5 mm MgCl_2_, and 3 mm DTT. For excitation, wavelengths of 546 nm and 360 nm were used for tamra and mant fluorophores, respectively, and a 570 nm (tamra) and 408 nm (mant) cut-off-filters (Schott glass) were, respectively, used to collect emitted light. To obtain high accuracy data, 4–6 measurements were performed and averaged. The observed rate constants (*k*_obs_) were fitted single exponentially using the GraFit program (Erithacus software).

##### Fluorescence Polarization

Experiments were performed in a Fluoromax 4 fluorimeter in polarization mode as described (46). Briefly, increasing amounts of different variants of IQGAP1 were titrated to mGppNHp- and tGppNHp-bound RHO proteins (1 μm) in a buffer containing 30 mm Tris/HCl, pH 7.5, 150 mm NaCl, 5 mm MgCl_2_, 3 mm DTT, and a total volume of 200 μl at 25 °C. For excitation, wavelengths of 546 nm and 360 nm (slit width: 8 μm) were used for tamra and mant fluorophores, respectively, and 579 nm (tamra) and 450 nm (mant) were used for emission (slit width: 10 μm). To increase the overall molecular mass of some IQGAP1 domains, GST fusion proteins were used to get larger increase in the polarization signal upon binding. The dissociation constants (*K_d_*) were calculated by fitting the concentration-dependent binding curve using a quadratic ligand binding equation.

##### Analytical Size Exclusion Chromatography

To show that the purified proteins are non-aggregating, analytical SEC was performed using a superdex 200 10/30 column (GE Healthcare). The buffer contained 30 mm Tris/HCl, pH 7.5, 150 mm NaCl, 5 mm MgCl_2_, and 3 mm DTT. For molecular mass determination the column was calibrated with standard proteins of known molecular mass: ferritin (440 kDa), aldolase (158 kDa), conalbumin (75 kDa), ovalbumin (43 kDa), carbonic anhydrase (29 kDa), and ribonuclease A (13.7 kDa). Protein samples (100 μm in 200 μl buffer) were injected onto the preequilibrated column, and the flow rate was sustained at 0.5 ml/min.

##### CD Spectroscopy

Far-UV CD spectra of IQGAP1 protein samples at protein concentrations ranging from 0.68 to 2.1 μm in 20 mm sodium phosphate buffer, pH 7.2, were recorded on a Jasco J-815 CD spectrometer (Jasco, Gross-Umstadt, Germany) equipped with a temperature-controlled cell holder running at 20 °C using a quartz cuvette with a 1-mm path length and instrument settings as follows: 0.2-nm step size, 50-nm min^−1^ scan speed, and 1-nm bandwidth. Signal-to-noise ratio was improved by accumulation of 10 scans per sample. The mean residue ellipticity [θ]_MRW_ in deg·cm^2^·dmol^−1^ was calculated from the equation [θ]_MRW_ = (θ_obs_ × MRW)/(*cd* × 10), where θ_obs_ is the observed ellipticity (in degrees), *c* is concentration (in g ml^−1^), *d* is cell path length (in cm), and MRW is the mean residue weight, (molecular weight divided by number of peptide bonds). Secondary structure fractions were calculated via the DiChroWeb program package (75) using the CDSSTR program (74)

##### Statistical Analysis

Data shown in the graphs are the average of triplicate or duplicate experiments. Data are expressed as the mean ± S.D. All stopped-flow experiments were performed in triplicate, and polarization experiments were performed in duplicate.

## Author Contributions

M.-R. A. conceived and coordinated the study. K. N., E. K. F., E. A., R. D., and L. G. designed, performed, and analyzed the experiments. K. N., E. K. F., E. A., D. J. T., D. W., L. S., and M. R. A. directed the experiments, analyzed the data, and co-wrote the paper. All authors reviewed the results and approved the final version of the manuscript.

## Supplementary Material

Supplemental Data

## Footnotes

3 The abbreviations used are:

GDI: guanine nucleotide dissociation inhibitor
WASP: Wiskott-Aldrich syndrome protein
IQGAP: IQ motif-containing GTPase activating protein
GRD: GAP-related domain
RGCT: RASGAP C-terminal domain
mant (m-): methylanthraniloyl
tamra (t-): tetramethylrhodamine
aa: amino acids
SEC: size exclusion chromatography
CT: C-terminal.
