# Supplementary material for: IQGAP1 Interaction with RHO Family Proteins Revisited: KINETIC AND EQUILIBRIUM EVIDENCE FOR MULTIPLE DISTINCT BINDING SITES
Source: J Biol Chem. 2016 Nov 4;291(51):26364–76. doi: 10.1074/jbc.M116.752121 (PMC5159498; doi:10.1074/jbc.M116.752121)
Supplement: Supplemental Data [file supp_291_51_26364__index.html]

IQGAP1 interaction with RHO family proteins revisited: Kinetic and equilibrium evidence for multiple distinct binding sites — IQGAP1 Interaction with RHO Family Proteins Revisited — New IQGAP Binding Mode for CDC42 and RAC1 — Supplemental Data 

# IQGAP1 Interaction with RHO Family Proteins Revisited

## Supplemental Data

- Supplemental Data (.docx, 1.4 MB) - JBC-2016-752121\_Revised Supplemental Information.docx
