# Supplementary material for: IQGAP1 Interaction with RHO Family Proteins Revisited: KINETIC AND EQUILIBRIUM EVIDENCE FOR MULTIPLE DISTINCT BINDING SITES
Source: J Biol Chem. 2016 Nov 4;291(51):26364–76. doi: 10.1074/jbc.M116.752121 (PMC5159498; doi:10.1074/jbc.M116.752121)
Supplement: Supplemental Data [file 10.1074_M116.752121_jbc.M116.752121-1.docx]

**Supplemental Information**

**IQGAP1 interaction with RHO family proteins revisited: Kinetic and equilibrium evidence for multiple distinct binding sites**

Kazem Nouri, Eyad K. Fansa, Ehsan Amin, Radovan Dvorsky, Lothar Gremer, Dieter Willbold, Lutz Schmitt, David J. Timson, and Mohammad R. Ahmadian

Institute of Biochemistry and Molecular Biology II, Medical Faculty of the Heinrich-Heine University, 40225 Düsseldorf, Germany.


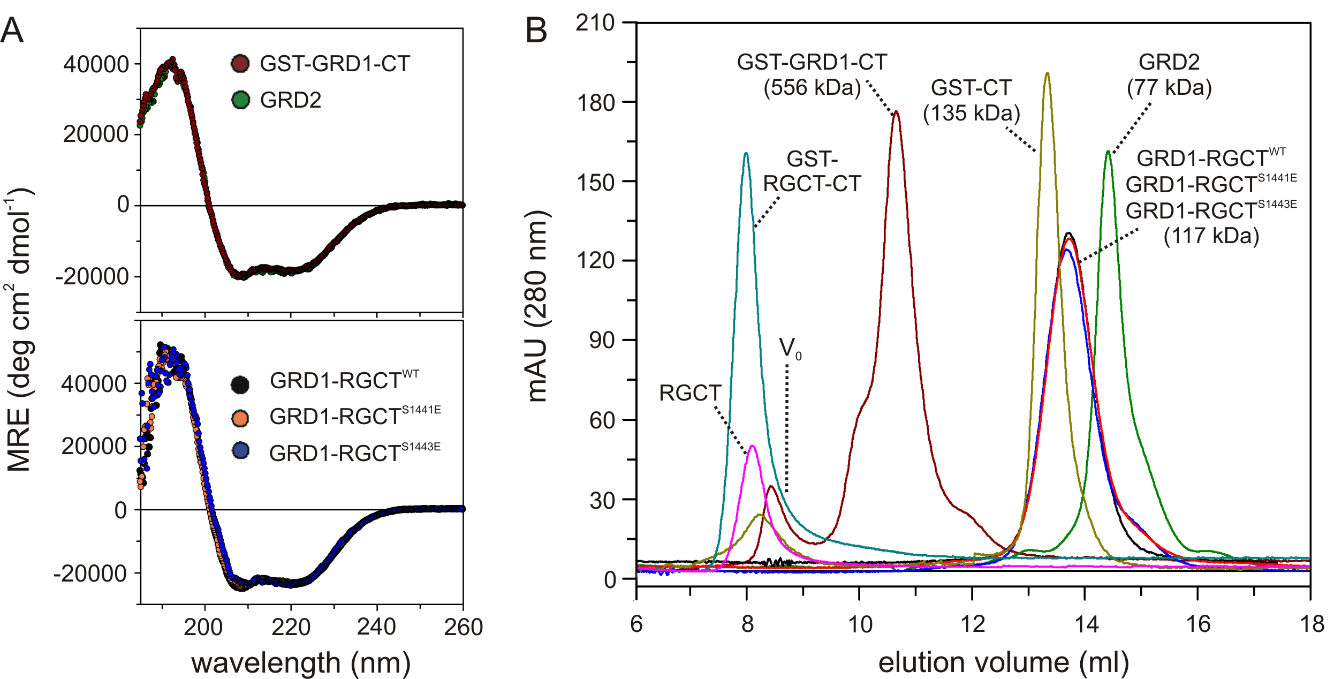


FIGURE S1. **Biophysical characterization of purified IQGAP1 variants.** **(A)** Circular dichroism (CD) measurements of various IQGAP1 variants. Obtained result showed that all proteins have almost a similar fold with ca. 60-75 % α-helices, 5-15 % β-strands, 5-10% turns, and 15-20% unordered. **(B)** Analytical size exclusion chromatogram of various IQGAP1 variants (as indicated) purified from *E. coli*. Void volume (V_0_) and elution volumes are shown on the x-axis. This column (Superdex 200, 10/300) was calibrated with molecular weight (MW) standards (see Experimental procedures). The chromatogram reveals `dimeric´ GRD2 (theoretical MW 42 kDa) and GRD1-RGCT variants (theoretical MW 75 kDa) with apparent MW of 77 and 117 kDa, respectively, and predominantly `tetrameric´ GST-GRD1-CT (theoretical MW 114 kDa) and GST-CT (theoretical MW 38 kDa) with apparent MW of 556 kDa and 135 kDa, respectively. RGCT-CT as GST-fusion and RGCT were eluted in V_0_. Tetramerization of GRD1-CT can be attributed to CT, as the latter also eluted as tetramer.

**Supplemental Information**

**IQGAP1 interaction with RHO family proteins revisited: Kinetic and equilibrium evidence for multiple distinct binding sites**

Kazem Nouri, Eyad K. Fansa, Ehsan Amin, Radovan Dvorsky, Lothar Gremer, Dieter Willbold, Lutz Schmitt, David J. Timson, and Mohammad R. Ahmadian

Institute of Biochemistry and Molecular Biology II, Medical Faculty of the Heinrich-Heine University, 40225 Düsseldorf, Germany.


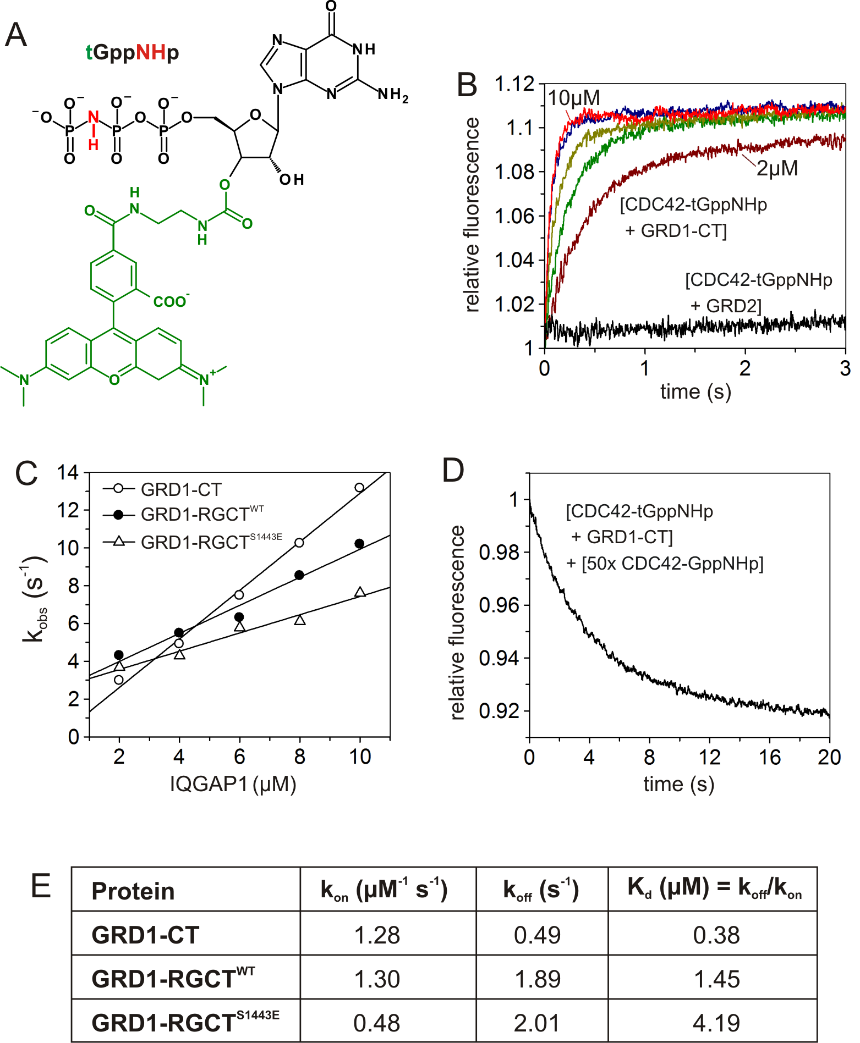


FIGURE S2. **Kinetics of interaction of CDC42-tGppNHp with GRD1-CT, GRD1-RGCT^WT^, and GRD1-RGCT^S1443E^.** **(A)** Chemical structure of tGppNHp, a non-hydrolyzable GTP analog, conjugated with fluorescent reporter tetramethylrhodamine (called tamra or t). **(B)** Association of CDC42-tGppNHp (0.2 μM) with increasing concentrations of GRD1-CT but not GRD2. **(C)** Association rate constant (k_on_) for GRD1-CT, GRD1-RGCTWT and GRD1-RGCTS1443E were evaluated from the plot of the k_obs_ values, obtained from the exponential fits to the association data in A, against the corresponding concentrations of the GRD1-RGCT variants. **(D)** Dissociation of GRD1-CT (2μM) from its complex with CDC42-tGppNHp (0.2 μM) in the presence of excess amounts of non-fluorescent CDC42-GppNHp (10 μM). **(E)** Summary of measured k_on_ and k_off_ values, and evaluated dissociation constants (K_d_). Obtained results with CDC42-tGppNHp are as compared to those obtained with CDC42-mGppNHp (see Fig. 5). All experiments were performed under the same conditions as in Fig. 5. The only difference was that tGppNHp was used instead of mGppNHp (see Fig. 2).

**Supplemental Information**

**IQGAP1 interaction with RHO family proteins revisited: Kinetic and equilibrium evidence for multiple distinct binding sites**

Kazem Nouri, Eyad K. Fansa, Ehsan Amin, Radovan Dvorsky, Lothar Gremer, Dieter Willbold, Lutz Schmitt, David J. Timson, and Mohammad R. Ahmadian

Institute of Biochemistry and Molecular Biology II, Medical Faculty of the Heinrich-Heine University, 40225 Düsseldorf, Germany.

#
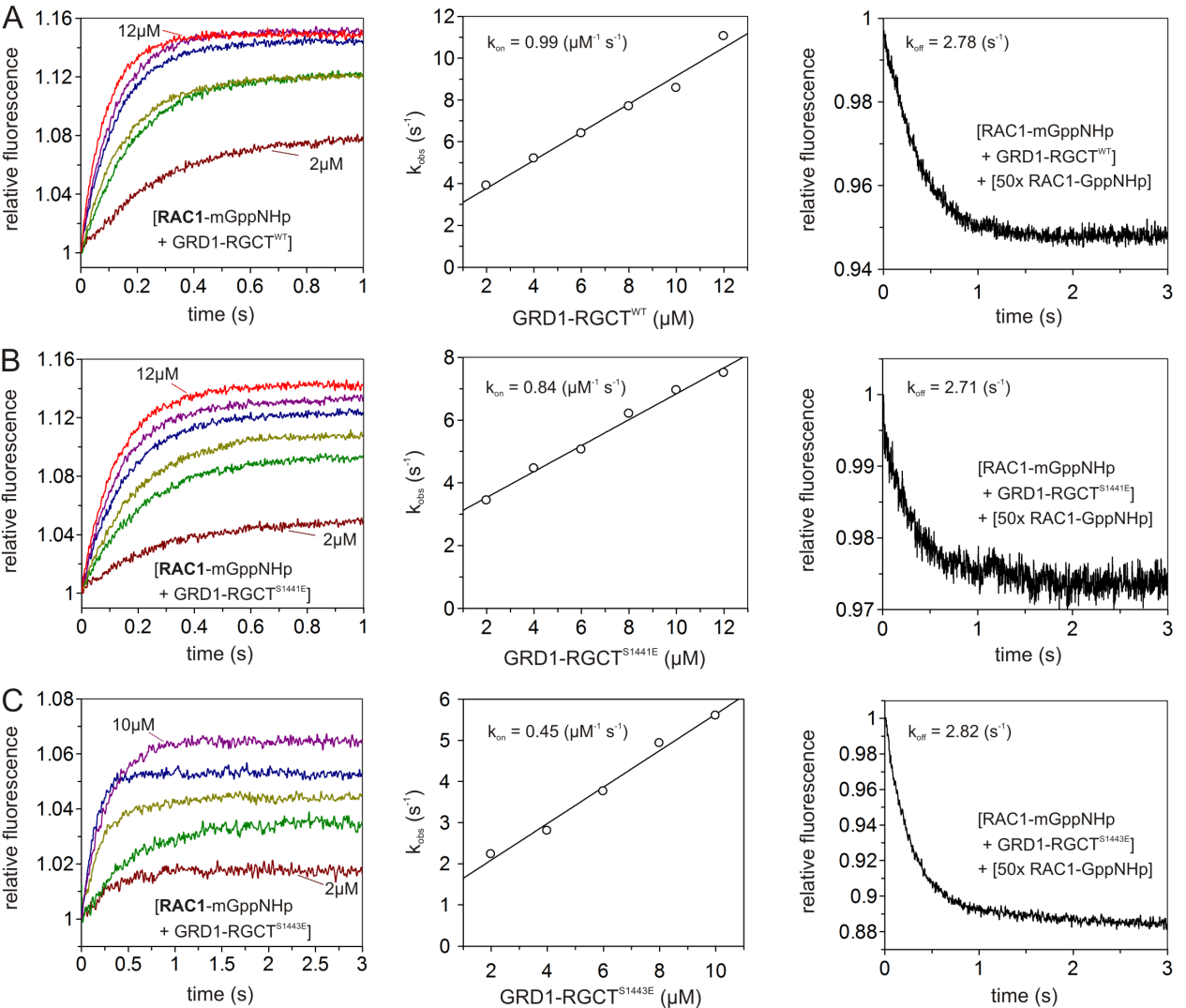


FIGURE S3. **Kinetics of interaction of RAC1-mGppNHp with different GRD1-RGCT variants, including WT (A), S1441E (B) and S1443E (C).** Left panels: Association of RAC1-mGppNHp (0.2 μM) with increasing concentrations of GRD1-RGCT variants. Middle panels: Evaluated association rate constant (k_on_) from the plot of the k_obs_ values, obtained from the exponential fits to the association data in the left panel, against the corresponding concentrations of the GRD1-RGCT variants. Right panels: Dissociation of the GRD1-RGCT variants (2 μM, respectively) from their complex with RAC1-mGppNHp (0.2 μM) in the presence of excess amounts of non-fluorescent RAC1-GppNHp (10 μM). Calculated dissociation constants (K_d_) are summarized in Figure 5C and Table 1.

**Supplemental Information**

**IQGAP1 interaction with RHO family proteins revisited: Kinetic and equilibrium evidence for multiple distinct binding sites**

Kazem Nouri, Eyad K. Fansa, Ehsan Amin, Radovan Dvorsky, Lothar Gremer, Dieter Willbold, Lutz Schmitt, David J. Timson, and Mohammad R. Ahmadian

Institute of Biochemistry and Molecular Biology II, Medical Faculty of the Heinrich-Heine University, 40225 Düsseldorf, Germany.

**
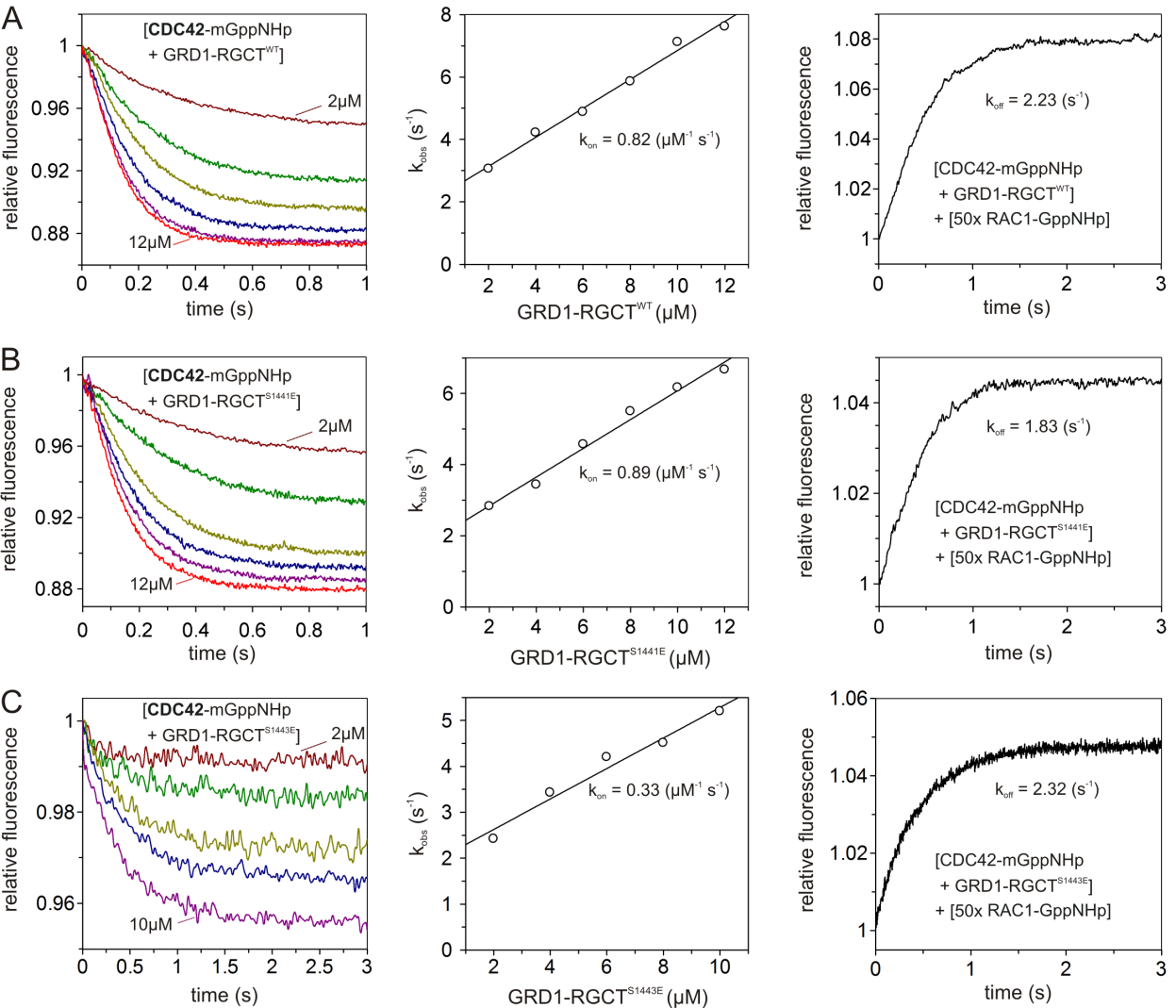
**

FIGURE S4. **Kinetics of interaction of CDC42-mGppNHp with different GRD1-RGCT variants, including WT (A), S1441E (B) and S1443E (C).** Left panels: Association of CDC42-mGppNHp (0.2 μM) with increasing concentrations of GRD1-RGCT variants. Middle panels: Evaluated association rate constant (k_on_) from the plot of the k_obs_ values, obtained from the exponential fits to the association data in the left panel, against the corresponding concentrations of the GRD1-RGCT variants. Right panels: Dissociation of the GRD1-RGCT variants (2 μM, respectively) from their complex with CDC42-mGppNHp (0.2 μM) in the presence of excess amounts of non-fluorescent CDC42-GppNHp (10 μM). Calculated dissociation constants (K_d_) are summarized in Figure 5C and Table 1.

**Supplemental Information**

**IQGAP1 interaction with RHO family proteins revisited: Kinetic and equilibrium evidence for multiple distinct binding sites**

Kazem Nouri, Eyad K. Fansa, Ehsan Amin, Radovan Dvorsky, Lothar Gremer, Dieter Willbold, Lutz Schmitt, David J. Timson, and Mohammad R. Ahmadian

Institute of Biochemistry and Molecular Biology II, Medical Faculty of the Heinrich-Heine University, 40225 Düsseldorf, Germany.

**
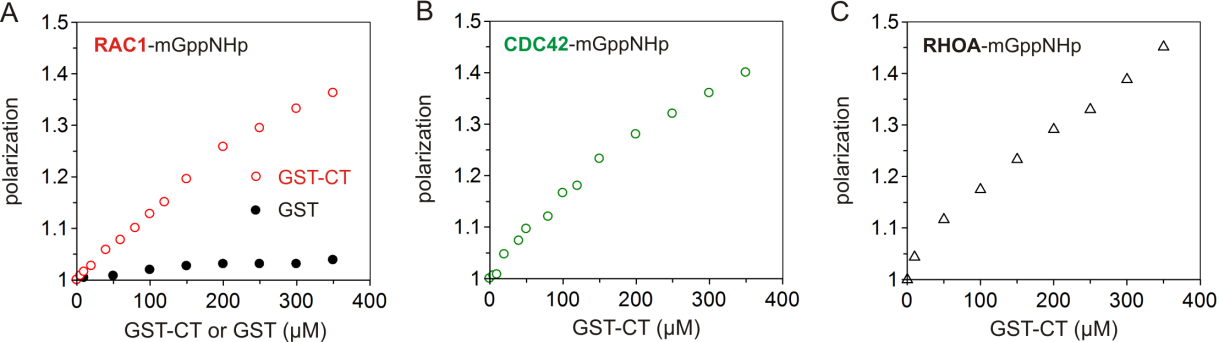
**

FIGURE S5. **A very weak binding of IQGAP1 CT to RAC1, CDC42, and RHOA proteins** **(A-C).** Fluorescence polarization experiments were performed using 1 µM mGppNHp-bound RAC1 **(A)**, CDC42 **(B)**, and RHOA **(C)**, respectively, and increasing concentrations of GST-CT (1 to 350 µM). Increased in polarization strongly suggest a very weak interaction between GST-CT with RHO proteins, which was not observed with GST alone (A).
